# Adrenal vein sampling: technique and protocol, a systematic review

**DOI:** 10.1186/s42155-021-00220-y

**Published:** 2021-04-01

**Authors:** Keith B. Quencer

**Affiliations:** Department of Radiology, Division of Interventional Radiology, University of Utah, 50 North Medical Drive, Salt Lake City, UT 84132 USA

**Keywords:** Primary aldosteronism, Aldosterone producing adenoma, Adrenal vein sampling, Cosyntropin

## Abstract

Primary aldosteronism is the leading cause of secondary hypertension worldwide. Its deleterious effects outstrip those due to blood pressure elevation alone. An essential part of the work-up of a patient with primary aldosteronism is determining if aldosterone production is unilateral or bilateral. With the former, surgery offers a potential cure and better overall outcomes. Adrenal vein sampling is considered the most reliable method to determine whether production is unilateral or bilateral. Sampling may be non-diagnostic when the vein cannot be properly cannulated. But with proper knowledge and experience as well as the utilization of certain techniques, procedure success can be high. Multiple protocols exist; their rationale and drawbacks are reviewed here. This article will give the reader an overview of techniques for improving procedural success as well as background, rationale and evidence to guide one in choosing the appropriate procedural and interpretation protocol.

## Background

The World Health Organization estimates that more than 1.3 billion people, nearly 31.3% of all adults, have hypertension (Mills et al. 2020). Hypertension is considered a leading cause of death and disability (Oparil et al. 2018). Primary aldosteronism (PA) is the most common cause of secondary hypertension, effecting an estimated 6% of all patients with hypertension and 20% of those with resistant hypertension (Byrd et al. 2018). Hyperaldosteronism causes hypertension via volume expansion due to sodium retention. Pathologic levels of serum aldosterone also exert pro-inflammatory and pro-fibrotic effects on the heart, blood vessels and kidneys (Brown 2013) leading to greater morbidity and mortality than essential hypertension, even when normalized for blood pressure elevation. This includes a 4.2x higher rate of stroke, 1.5x higher rate of renal damage, 2.6x higher rate of myocardial infarction and 5x the rate of atrial fibrillation (Milliez et al. 2005; Rossi et al. 2006; Rossi et al. 2013; Savard et al. 2013).

In current clinical practice, the diagnosis of primary hyperaldosteronism occurs in 2 stages. In properly selected patients (Table 1), screening is performed by measuring serum aldosterone and renin. The aldosterone: renin ratio (ARR) is then calculated. Values > 20 are consistent with PA. Absolute values should also be evaluated as very low renin levels will potentially lead to a false positive test by exaggerating the ARR even in the setting of low aldosterone levels (Maiolino et al. 2017). After a positive screening test, confirmatory tests are often performed. Serum or urinary aldosterone levels are measured after sodium loading; persistent elevation confirms autonomous aldosterone secretion.

**Table 1:** Indications for screening for PA

Resistant Hypertension*	
Hypertension with a family hx of PA	
Hypertension with a family hx of early onset HTN or stroke (<40y/o)	
Hypertension with hypokalemia	
Hypertension with adrenal adenoma	
Hypertension with obstructive sleep apnea^	

Guideline recommendations for screening of patients for primary aldosteronism. (Funder et al. 2016): *- Sustained BP > 150/100 mmHg without treatment, > 140/90 mmHg on 3 antihypertensive drugs or < 140/90 mmHg but requiring at least 4 antihypertensive drugs. In one study, 33.9% of patients with obstructive sleep apnea were found to have PA (Di Murro et al. 2010)

The next step, termed subtyping, is determining if production is unilateral or bilateral. In the former case, an aldosterone producing adenoma (APA) is the most likely cause with less likely etiologies being diffuse or nodular unilateral hyperplasia. Unilateral aldosterone production is most effectively treated with adrenalectomy. Bilateral production is termed idiopathic hyperplasia. Bilateral production is treated medically, utilizing mineralocorticoid receptor antagonists and, if needed, potassium sparing diuretics like amiloride. Spironolactone is a non-selective mineralocorticoid receptor (MR) antagonist which has anti-adrenergic effects potentially leading to gynecomastia and/or other sexual side effects. A more selective MR antagonist, eplerenone, can be used when spironolactone is not tolerated.

## Main text

The recommended technique for lateralization of aldosterone production is adrenal vein sampling (AVS) (Funder et al. 2016; Rossi et al. 2014). The indications, protocols, techniques and outcomes are the subject of this review. For this manuscript, studies and trials published on adrenal vein sampling since 2015 were searched in PubMed. Twenty-one articles pertaining to technique and protocol during this time period were reviewed. Additionally, a search was performed for consensus guidelines and expert opinions since 2015. Three were reviewed and incorporated into this review. Additional articles published outside this time period were reviewed and cited, if relevant.

Computed tomography (CT) and AVS have a high rate of discordance in subtyping patients with PA. A meta-analysis in 2009, (Kempers et al. 2009), which included 38 studies and 950 patients, showed only a 62.2% concordance between cross sectional imaging and adrenal vein sampling (Table 2). The reasons for this discordance are legion. Cross sectional imaging (CT or magnetic resonance imaging-MRI) is insensitive in detecting sub-centimeter adrenal adenomas, which make up the majority of APAs. Additionally, both CT and MRI are insensitive to detect pathologically proven unilateral hyperplasia, which in one study was found to make up to 45% of cases unilateral production (Citton et al. 2015). Cross sectional imaging can be non-specific. Incidentally discovered adrenal adenomas are prevalent, seen by cross sectional imaging in about 1.9% of patients (Sherlock et al. 2020), the majority (≈90%) of which are considered non-functional. This relatively high prevalence of incidental adrenal adenomas limits the specificity of CT, especially in older patients who have a higher incidence of these adenomas (Kloos et al. 1997). Given the rarity of incidental adrenal lesions in young patients, the widely adopted Mayo Clinic protocol makes an exception to the recommendation for use of AVS to subtype PA in patients. Patients < 40 years old who have a unilateral adrenal adenoma > 1 cm and a normal contralateral gland may proceed directly to adrenalectomy (Kupers et al. 2012). This exception has been called into question. For example, Citton et (Citton et al. 2015) al showed 2 failures of biochemical cure in patients < 40 who underwent adrenalectomy based on cross sectional imaging alone. Ladurner (Ladurner et al. 2017) showed a 9.5% (2/21) CT and AVS discordance in the under 40 subset of their study.

**Table 2:** Discordance between AVS and cross sectional imaging according to meta-analysis performed in 2009 which included 38 studies and 950 patients. (Kempers et al. 2009). In cases where CT is non-lateralizing (either because both glands are normal or both glands are abnormal) but AVS is lateralizing, patients would have been inappropriately excluded from potentially curative surgery. In cases where CT suggests unilateral production but AVS shows bilateral production, patients would be subjected to inappropriate surgery. Finally, in cases where CT demonstrates unilateral pathology but AVS demonstrates contralateral pathology, these patients would have undergone wrong side surgery

Concordance	CT bilateral, AVS unilateral	CT unilateral, AVS bilateral	CT and AVS unilateral but opposite
62.2% (591/950)	19.2% (182/950)	14.7% (140/950)	3.9% (37/950)

The proportion of patients with PA who have unilateral production determined by AVS differs by referral pattern and criteria used and is typically between 1/3rd and 2/3^rds^ of patients. Patients with more severe PA phenotype (i.e. spontaneous hypokalemia and/or higher aldosterone levels) are more likely to have an APA and therefore have unilateral production. While unilateral production can be effectively treated medically, patients who undergo AVS directed adrenalectomy have a high rate of biochemical cure (Table 3), a high rate of resolution of hypokalemia, potential for hypertension cure (≈35%), lower hypertensive medication need, less medication side effects and overall better quality of life (Rossi et al. 2019). Higher rates of hypertension cure after adrenalectomy are seen in patients with short duration hypertension, younger age, normal renal function female gender and normal body mass index (Funder et al. 2016; Rossi et al. 2014).

**Table 3:** Rate of biochemical PA cure cure after AVS guided adrenalectomy

Study	Rate of biochemical cure
Umakoshi (Umakoshi et al. 2018)	81.0% (187/231)
SPARTICUS (Dekkers et al. 2016)	88.1% (37/42)
AVIS-2 (Rossi et al. 2019)	93.8% (487/519)
Laurder (Ladurner et al. 2017)	95.9% (142/148)
Lim (Lim et al. 2014)	96.5% (112/116)
Citton (Citton et al. 2015)	100% (56/56)
Pasternak (Pasternak et al. 2016)	100% (45/45)

The SPRATICUS study (Dekkers et al. 2016) called into question need for AVS to subtype PA. Patients were triaged to surgical or medical treatment based either on AVS or CT; there were similar overall clinical results with no significant difference in the primary endpoint, the daily defined dose, which is a validated measure of total consumption of antihypertensive drugs to control blood pressure. Secondary endpoints such as hypertension cure and biochemical cure were not statistically different between the two groups but trends favoring he AVS arm were seen in these two outcomes. After adrenalectomy, biochemical persistence was seen in 5/46 patients (11%) of the AVS subgroup compared to 9/46 (20%) in the CT subgroup. Hypertension cure was seen in 10/46 (22%) in the AVS arm but only 4/46 (9%) in the CT arm. The study, however, was not powered to evaluate these secondary endpoints. This study had balanced randomization and had 92% (184/200) 1-year clinical follow-up. Criticisms of the study included high percentage (68%) of patients in the study with hypokalemia, which only occurs 9–37% of all patients with PA (Funder et al. 2016), which may limit the generalizability of the study. As aldosterone producing adenomas are seen in about 50% of PA patients with hypokalemia compared with only 20% of patients without hypokalemia, CT may be of greater utility in this more selected patient population who may have more APAs visible by CT (Rossi and Funder 2017). In this study, patients who underwent AVS also underwent CT as part of standard of care. Similar to results of multiple prior studies (see Table 2) there was a high rate of discordance between AVS and CT in SPARTICUS. That final clinical outcomes were similar between the two arms despite the high rate of discordance underlies potential issues and areas for improvement in subtyping both by CT and AVS.

Adrenal vein sampling may be non-diagnostic in a significant proportion of cases (Table 4). One study that highlighted this difficulty was the German Conn registry which included a total of 306 patients undergoing AVS. Diagnostic adequacy was achieved in only 41.1% of procedures (Vonend et al. 2011). But, with proper training, knowledge and experience it can be performed successfully Other studies since have shown a higher success rate. AVS is typically done at major referral centers (Rossi et al. 2014) and has a greater success in the hands of interventionalists experienced in this procedure. The learning curve is estimated to be about 20–30 cases with maintenance of proficiency of about 15 annual cases (Jakobsson et al. 2018). In low volume centers, it may therefore be necessary to restrict AVS performance to a single dedicated interventional radiologist.

**Table 4:** Success and Failure Rate of Recently Published AVS studies

Study	Success rate	Failure R only	Failure L only	Failure B
German Conn (Vonend et al. 2011)	41.1 (126/306)	36.6 (112/306)	9.5 (29/306)	12.7 (39/306)
Deipolyi (Deipolyi et al. 2015)	63 (58/92)	30.4 (28/92)	2.1 (2/92)	4.3 (4/92)
Teng (Teng et al. 2015)	55% (26/47)	NR	NR	NR
Page (Page et al. 2018)	71.4% (105/147)	23.1% (34/147)	5.4% (8/147)	0/147
Kocjan (Kocjan et al. 2020)	77% (198/259)*	14%(33/235)*	3.8% (9/235)*	NR
AVIS-2 (Rossi et al. 2019)	80.1% (1302/1625)	NR	NR	NR
Lee (Lee et al. 2020)	89.5% (43/48)	6.3 (3/48)	0% (0/48)	4.2% (2/48)
Sparticus (Dekkers et al. 2016)	95.8% (92/96)	4.2% (4/96)	0	0
Ota (Ota et al. 2016)	99.2% (124/125)	0.8% (1/125)	0	0

AVS success rates of published articles in last 10 years. *-This study variably reported success rates by procedure for overall and then by patient regarding left or right side. As 10% of patients had a repeat AVS, the reported right and left success rates differ from overall success. NR = not reported. Success rates depend on the strictness of criteria used to define success (Lethielleux et al. 2015), which were not standardized across these studies

Nuclear medicine studies utilizing, including Iodine-131 6-beta-iodomethyl-19-norcholesterol scintigraphy (NP-95) (Wu et al. 2019) and positron emission tomography imaging utilizing 11beta-hydroxylase 11C-metomidate (MTO) (Bergstrom et al. 2000) are cumbersome to perform and have limited accuracy. They are not utilized in current clinical practice.

### Patient preparation

Prior to AVS, all patients should undergo cross sectional imaging. This can rule out the rare cases of PA caused by adrenal cortical carcinoma, in which case adrenalectomy should be performed without the need for AVS. CT is preferred over MRI for three main reasons: 1. CT better characterizes fat poor adenomas based on washout calculations (Seo et al. 2014), 2. CT has better spatial resolution and can better visualize small adrenal lesions, 3. It is more advantageous in pre-procedural planning. CT visualizes the right adrenal vein in 93.2% of cases compared to 84.8% in MRI (Ota et al. 2016). This not only helps direct where to search during AVS but also identify anatomic variants, such as communication between the right adrenal and hepatic veins (Matsuura et al. 2008).

All patients should be willing surgical candidates prior to AVS. One study showed a 21.8%% rate of patients lateralized by AVS who do not undergo surgery. AVS in these patients was a misuse of resources and was unnecessarily invasive (Ohno et al. 2020). Additionally, if there is a suspicion of familial PA, based on young age of onset, a strong family history of PA or a family history of strokes at a young age, types I and III should be ruled out prior to AVS as adrenalectomy is not indicated because bilateral secretion is the rule in these patients.

Drugs that interfere with the renin- angiotensin-aldosterone system including angiotensin converting enzyme inhibitors, angiotensin receptor blockers, and mineralocorticoid receptor antagonists should be discontinued. As these drugs increase renin levels, bilateral adrenal cortex stimulation would occur, falsely decreasing the rate of lateralization. Literature is variable about the length of time these medications should be discontinued. Some suspend them for 2 weeks (Deipolyi et al. 2015), others discontinue these for 8 weeks (Lee et al. 2020) and still others also assess for suppressed plasma renin activity (< 0.6 ng/ml/hr) prior to proceeding with AVS (Kocjan et al. 2020). Doxazosin, hydralazine, diltiazem and nifedipine can be used as substitute antihypertensive agents. Patients should be told to institute an unrestricted sodium diet as low sodium diets lead to bilateral aldosterone secretion. Because discontinuing mineralocorticoid receptor blockers may lead to hypokalemic recidivism, serum potassium should be measured and, if < 3.5 mmol/L it should be corrected; low potassium inhibits aldosterone production and increases the proclivity of arrhythmias.

Pre-procedure clinic consultation with the interventionalist should be considered to ensure proper medications are held, potassium levels are checked and adequate replacement antihypertensives are being used. During this visit, personal and published rates of non-diagnostic studies and complications should be reviewed. If possible, the procedure should be scheduled in the morning when natural cortisol levels are highest.

### Procedure general

There are many variations in how AVS is performed (Table 5).

**Table 5:** Marked heterogeneity of basic procedural aspects of AVS

Study	Cosyntropin use	Sequential v simultaneous	Selectivity index	Lateralization index
SPARTICUS (Dekkers et al. 2016)	Cont. infusion	sequential	≥3	≥4.0 w/ CSI ≤1
Deipolyi (Deipolyi et al. 2015)	Cont. infusion	sequential	˃3	˃4
Kocjan (Kocjan et al. 2020)	Cont. infusion	sequential	˃5	˃4
Pasternak (Pasternak et al. 2016)	Cont. infusion	sequential	NR	≥4
Ota (Ota et al. 2016)	Bolus 250 μg, sample 15 min later	Simultaneous	≥5	NR
Miotti (Citton et al. 2015; Miotto et al. 2009)	No stim	Simultaneous	≥1.1	≥2.0
Conn (Vonend et al. 2011)	No stim	Sequential	≥2	≥3
Wolley (Wolley et al. 2015)	No stim	Sequential	≥3	≥2^ with CSI ≤1
Lee (Lee et al. 2020) Bellavance (Bouchard-Bellavance et al. 2020)	Pre and post 250mcg bolus	Simultaneous	≥3 Pre ≥5 Post	> 2 pre cosyntropin > 4 post cosyntropin
Teng (Teng et al. 2015)	Pre and post (bolus + infusion)	Sequential	> 2 Pre cosyntropin > 3Post cosyntropin	> 3 Pre cosyntropin > 4 Post cosyntropin
Webb (Webb et al. 2012)	Pre and post (bolus + infusion)	Simultaneous	> 5	> 4
Japanese Endocrine (Nishikawa et al. 2011)	250 μg cosyntropin bolus	Simultaneous	≥5 and ≥ 200 μg/dl cortisol	≥2.6 or unilateral aldosterone ≥14,000 pg/ml
Funder (Funder et al. 2016)	No conclusive rec	No conclusive rec	> 5 with cosyntropin > 2 without cosyntropin	> 4 with cosyntropin > 2 without cosyntropin
Expert Consensus (Rossi et al. 2014)	No conclusive rec	No conclusive rec	≥3 with cosyntropin ≥2 without cosyntropin	≥4 with cosyntropin ≥2 without cosyntropin

NR = not reported. ^^^Wolley et al. used a modified lateralization index of (Dominant Adrenal Aldosterone/Cortisol)/(IVC Aldosterone/Cortisol) ≥2 and a CSI ≤1

### Cosyntropin Use

One fundamental difference is whether synthetic adrenocorticotropic hormone (ACTH), known as cosyntropin, is used. The rationale for using cosyntropin is to increase cortisol secretion as well as to stabilize temporal fluctuation of both aldosterone and cortisol (Rossitto et al. 2020). After ACTH administration, adrenal vein cortisol levels increase by a factor of 5-10x while peripheral cortisol levels remain relatively stable. This increased step-up between adrenal and peripheral cortisol increases a ratio known as the selectivity index (SI) which is used to aver sampling adequacy (Table 6). Cosyntropin leads to a 4x increase in procedural “success”, even when increasing the SI threshold from 2➔ 5 (El Ghorayeb et al. 2016). A subset of patients in the Adrenal Vein International Study (AVIS)-2 study underwent AVS both pre and post cosyntropin stimulation, In these patients, there was significantly higher sampling adequacy (81.3%), even using the strictest SI (≥5), when compared to only 67.3% sampling adequacy in unstimulated patients with a low SI (≥2) (Rossitto et al. 2020). Additionally, cosyntropin may increase adrenal blood flow thereby enlarging the adrenal veins and making cannulation easier (Violari et al. 2019).

**Table 6:** Commonly used formulas in adrenal vein sampling

Description	Formula	Use
Selectivity Index (SI)	“adrenal vein” cortisol/peripheral vein cortisol	Ascertain if sampling was adequate
Lateralization Index (LI)	(dominant adrenal vein aldosterone÷cortisol)/ (non-dominant adrenal vein aldosterone÷ cortisol)	Determine if production is unilateral or bilateral
Contralateral Suppression Index (CSI)	(Non-dominant adrenal vein aldosterone ÷ cortisol)/ (peripheral aldosterone÷peripheral cortisol)	Adjunct value to determine if production is unilateral or bilateral

Cosyntropin use is not without potential disadvantages; it stimulates normal adrenal gland aldosterone production but has a varied, and sometimes minimal, effect on APA aldosterone production. This may lead to falsely non-lateralizing studies. There is a 22–25% increase in “bilateral” diagnoses in stimulated samples compared to unstimulated samples (Violari et al. 2019) (Teng et al. 2015). In both of these studies, patients were triaged to surgery based on pre-ACTH sampling and, even in patients whose post-ACTH was no longer lateralizing, biochemical cure was the rule. In the AVIS-2 study (Rossitto et al. 2020), 402 of their 1625 patients (24.7%) had pre and post stimulation samples drawn. The authors estimated that, depending on exact indices used, approximately 32% of results would change from unilateral on pre-stimulation to bilateral on post stimulation. Cosyntropin may lead to other altered results. A study which performed sampling first without and then with a bolus of 250 μg of cosyntropin highlighted this. A total of 28% (44/157) of patients had a different results comparing unstimulated and stimulated samples. While the majority (72.7% (32/44)) of these changes were lateralized without but bilateral with stimulation, 20.5% (9/44) changed from being bilateral on basal study to being unilateral after stimulation and 6.8% (3/44) of cases lateralized to the opposite side and (El Ghorayeb et al. 2016).

### Procedural technique

Right adrenal vein cannulation is the most difficult part of AVS; failure to cannulate the right adrenal vein is the most common cause of an unsuccessful procedure (see Table 4).

A solid understanding of the anatomy of the right adrenal vein is necessary for successful cannulation. It is small in diameter (2-4 mm) and short and most often enters the IVC at the T11/T12 interspace from a posterior-lateral direction. Other more subtle issues surrounding right adrenal vein anatomy are important to be aware of but are variably described in the literature. There are conflicting reports of the presence or absence of multiple adrenal veins. One study, which included 170 patients undergoing laparoscopic adrenalectomy for hyperaldosteronism, showed 4 duplications (2.4%) and 1 triplication (0.6%) (Scholten et al. 2013). This is in distinction to another study, utilizing CT (Matsuura et al. 2008), that showed no evidence of multiple adrenal veins (0/79). If multiple adrenal veins are present, AVS may be misleading if the vein draining effluent from an APA is not sampled. This should be suspected if cortisol levels show the samples to be adequate but both adrenal veins show suppressed corrected aldosterone levels when compared with peripheral venous blood.

Another basic yet controversial anatomic topic is whether the right adrenal vein can share a common trunk with an accessory hepatic vein. In 800 AVS cases, Durant reported no venographic evidence of a common trunk between the adrenal vein and accessory hepatic veins. Communication between the adrenal vein and hepatic veins was seen by small capsular/superficial communicating veins in 0.25% (2/800) patients (Daunt 2005). On the other hand, other studies report a relative high frequency of direct communication between an accessory hepatic vein and the right adrenal vein. Matsuura (Matsuura et al. 2008) reported a 8% (6/79) incidence by CT, Miotto (Miotto et al. 2009) reported a 12.1% (8/66) incidence by venography, and Ota (Ota et al. 2016) found a 16% (20/125) incidence by MR, CT and/or venography.

Multiple different catheters can be used to select the right adrenal vein. Caution is advised when using a reverse curved (e.g. Sim-2) catheter (Tan et al. 2020; Zelinka et al. 2012) as this may lead to too deep of cannulation, potentially beyond a tributary draining an APA. Deep cannulation can also increase the propensity for vein rupture or venous infarct.

**Fig. 1:**
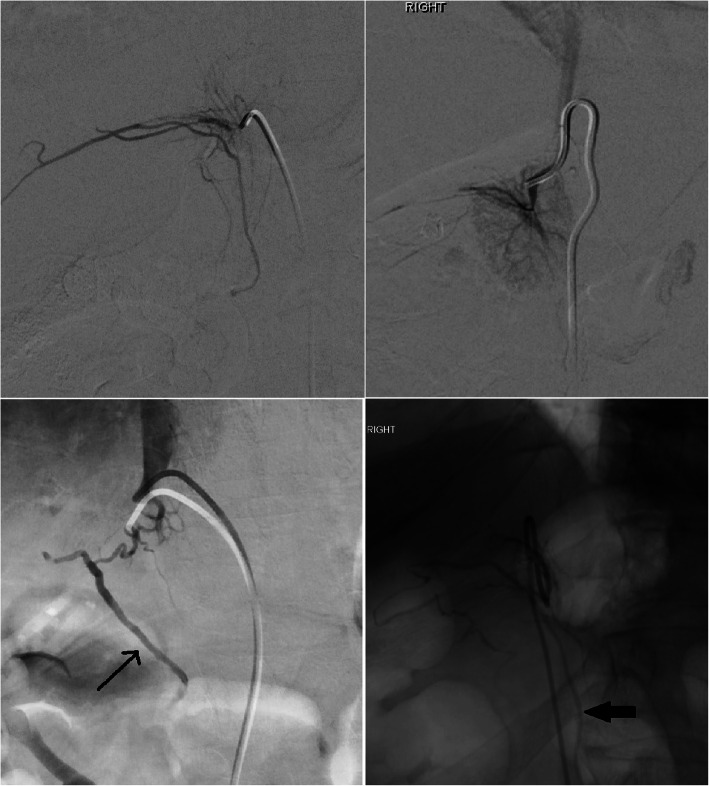
Varied appearance of the right adrenal vein in 4 different patients. All 4 images are of the right adrenal vein, confirmed by elevated cortisol levels. Images **c** and **d** show an inferior emissary vein (black arrows), diagnostic of adrenal vein cannulation

**Fig. 2:**
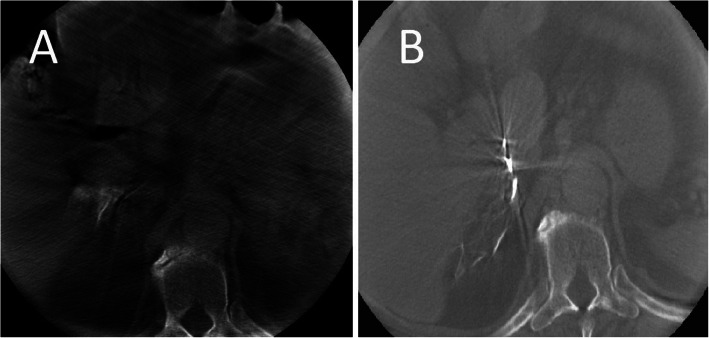
Cone-beam CT of an accessory hepatic vein (**a**) and the right adrenal vein (**b**). In this patient, two different veins were cannulated. Given ambiguous conventional venographic appearance, cone-beam CT was performed. **a** Shows opacification of hepatic parenchyma. **b** Confirms cannulation of the adrenal vein

**Fig. 3:**
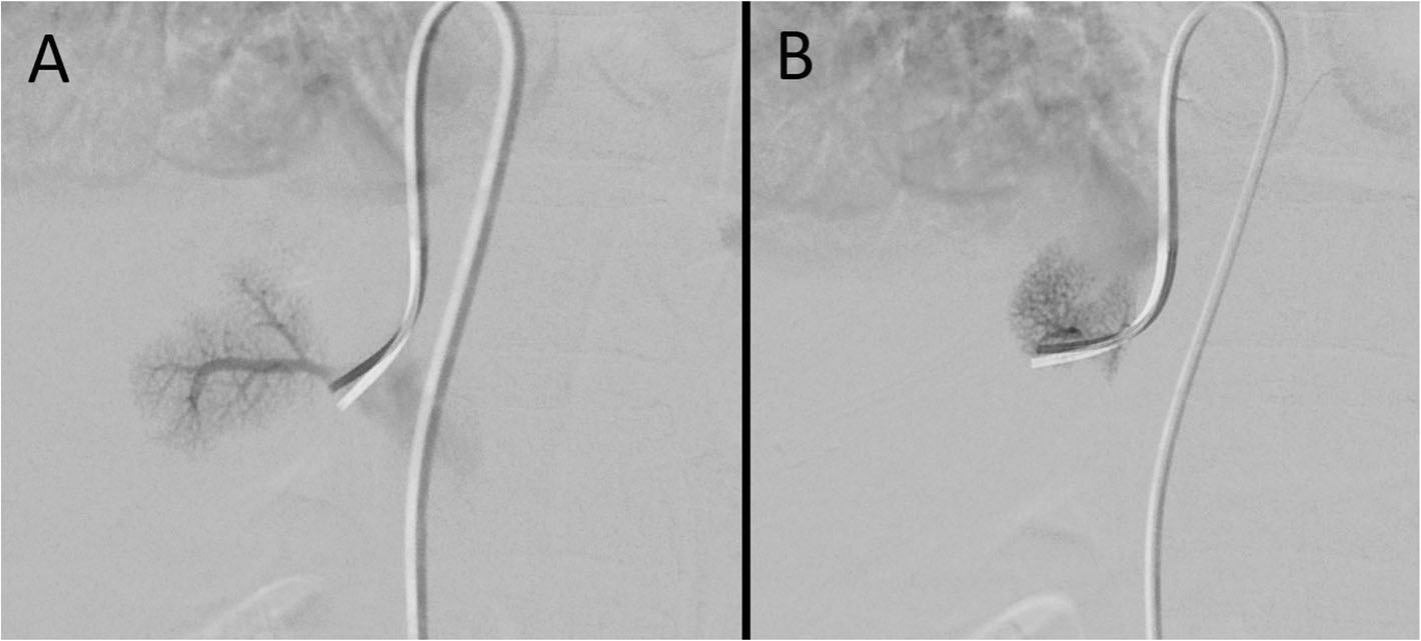
Venographic appearance of an accessory hepatics vein. Images A + B, taken in the same patient show two different accessory hepatic veins. The parenchymal staining and lack of capsular/communicating veins are consistent with hepatic vein rather than adrenal vein cannulation. Occasionally, one may see intrahepatic communication to larger hepatic veins

Once a candidate right adrenal vein is cannulated, venogram is performed. Care must be taken to perform only gentle, slow and low volume injection to avoid rupture of the fragile adrenal veins. The adrenal gland has a varied venographic appearance (Fig. 1) with the only pathognomonic finding being an inferior emissary vein. The most common appearance of the right adrenal vein is a tangle of spidery vessels at the expected location of the adrenal gland. Capsular and superficial communicating veins communicating with phrenic, intercostal or renal capsular veins are common. In cases of ambiguous venographic appearance, rotational CT may be helpful (Deipolyi et al. 2015; Kocjan et al. 2020) (Fig. 2). Small accessory hepatic veins have a similar appearance but certain characteristics are useful to distinguish them from the adrenal vein (Fig. 3). Key among them is that injection of hepatic veins can lead to hepatic parenchymal staining, which is rarely seen in adrenal venography. This hepatic sinusoidal staining occurs without patient symptoms whereas, if one injects hard enough in an adrenal vein to cause an adrenal parenchymal stain, vague chest, flank or abdominal discomfort may occur.

After deciding the cannulated vein likely represents an adrenal vein, one should proceed with sampling. Gentle, intermittent or gravity assisted aspiration is preferred over high negative pressure aspiration which may collapse the adrenal vein and prevent venous sampling. The first 2 cc of aspirated fluid should be wasted as iodinated contrast has been shown to interfere with measurement of serum aldosterone levels. A side hole placed 3 mm from the tip of the catheter is generally recommended to improve flow through the catheter. This may also decrease the risk of venous thrombosis as the catheter is less occlusive within the adrenal vein. One may also place a 0.018″ wire through the 5Fr catheter to allow for centering of the catheter within the right adrenal vein; aspiration is then done through the side arm of a rotating hemostatic valve (Mailhot et al. 2015). Confirmatory venogram after sampling is recommended to ensure that the catheter has not changed positions.

### Left adrenal

**Fig. 4:**
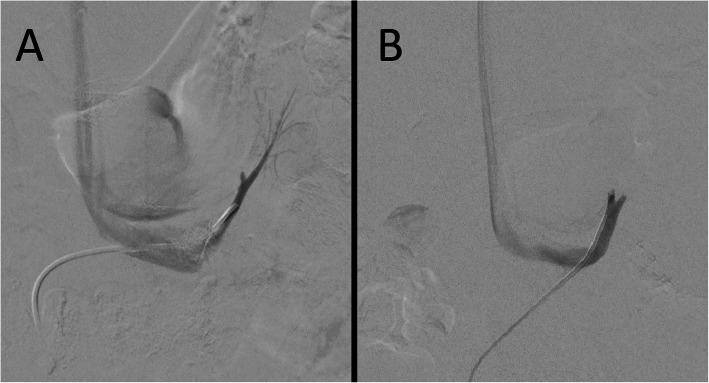
Venographic appearance of the left adrenal vein. In both A + B a Simmons-2 catheter is used to select the left renal vein and then the common phrenic adrenal trunk. In **a**, contrast refluxes into the adrenal branch (lateral). In **b**, the common trunk and a small part of the inferior phrenic vein (medial) and the left adrenal vein (lateral) are seen. Reflux of contrast into the adrenal vein proper is not necessary to confirm correct location

In sequential AVS, the right adrenal vein cannulation, which is more time consuming than the left, should be done first thereby decreasing the time gap between samplings. The left adrenal vein has a constant anatomic position and joins the inferior phrenic vein to form a variable length phrenic adrenal trunk before entering the cranial aspect of the left renal vein (Fig. 4). There are many catheters and methods of left adrenal vein; one simple way is to select the left renal vein using a Simmons-2 catheter. The catheter is then pulled down, initially causing the tip to enter further into the left renal vein. Eventually, further catheter retraction will start pulling the catheter back and the catheter tip will “jump” up just lateral to the spine to engage the phrenic adrenal trunk.

Most operators sample from this common trunk. Some, including the Japanese endocrine society, advocate for selective engagement the left adrenal vein branch (Kocjan et al. 2020; Lupi et al. 2019; Nishikawa et al. 2011; Ota et al. 2016; Rossi et al. 2014). But, selective sampling may lead to accidental sampling beyond an aldosterone rich tributary, adds cost to the procedure given the need for a microcatheter and microwire). Additionally, sampling in the left adrenal vein proper may predispose to thrombosis or to vessel rupture during injection. One small study of 22 patients (Takada et al. 2013) compared sampling from the adrenal vein proper and the common phrenic adrenal trunk. While absolute aldosterone and cortisol levels increased, the A/C ratio and overall results did not significantly change. Therefore, sampling in the common trunk is recommended.

### Interpretation of results (Table 7)

**Table 7:** Results from AVS in a 31y/o female with a history of hypertension and hypokalemia who was found to have PA by ARR (237). CT (not shown) showed normal appearing bilateral adrenal glands. Samples were taken with continuous cosyntropin infusion at 50 μg/hr. Left sided sampling was done in the common phrenic adrenal trunk. Ratios between the adrenal vein cortisol and IVC cortisol, the SI, are used to determine sampling adequacy. In this case SI on the right is 38.1 (914/24) and 6.6 (159/24) on the left, the latter number lower because of dilution from the inferior phrenic vein. The lateralization index is 20 (7.2/.35) with a CSI of 0.16 (.35/2.2). The patient underwent left adrenalectomy with hypertension and biochemical cure

	Aldosterone (ng/dl)	Cortisol (μg/dl)	A/C ratio
Right Adrenal Vein	319	914	0.35
Left Adrenal Vein	1144	159	7.2
Inferior Vena Cava	52	24	2.2

### Adequacy

As cortisol is produced solely by the adrenal glands, cortisol gradient between the peripheral veins and “adrenal” vein is used to confirm adequate adrenal vein cannulation. There is a wide variety (≥1.1 to ≥5) of selectivity indices used to determine sampling adequacy (see Table 5). Selectivity indices > 2 and > 5 are generally used as cutoffs for adequacy with unstimulated and stimulated sampling, respectively. These existing guidelines have been called into question. The AVIS-2 analysis (Rossitto et al. 2020) showed similarity of specimen adequacy with unstimulated SI of ≥1.4 and stimulated SI of ≥5 suggesting that a more permissive SI for unstimulated samples may be indicated.

### Lateralization

Lateralization indices of > 2 for unstimulated and > 4 for stimulated sampling are recommended by the endocrine society (Funder et al. 2016). One study of stimulated AVS (Umakoshi et al. 2018), showed that biochemical cure was only achieved in 64.2% (29/47) of patients with an LI between 2 and 4 but a 80.9% (187/231) biochemical cure in patients in patients with LI > 4. The PASO study (Williams et al. 2017) and a multicenter Japanese study (Umakoshi et al. 2018) also showed that higher LIs correlated with significantly higher clinical and biochemical success rates after adrenalectomy. The contralateral suppression index may be helpful adjunctive lateralizing data. One study (Wolley et al. 2015) showed that 82.5% (66/80) of patients who lateralized by LI also had concurrent CSI < 1. This subset of patients had a higher rate of hypertension and biochemical cure (40.9%; 27/66 and 98%; 48/49) respectively compared to those who met traditional LI criteria but whose CSI was > 1 (14.3%; 2/14 and 55.6% 5/9). As medical therapy is effective in cases of unilateral disease but surgery is ineffective in bilateral disease, meeting the additional condition of contralateral supression prior to adrenalectomy may be prudent.

### AVS limitations

AVS is only offered at a limited number of referral centers making it relative inaccessible and underutilized. Missed opportunities for adrenalectomy or non -AVS guided adrenalectomy often occur (Funder 2012; Rossi 2007). In the AVIS-2 study, 23.6% (160/679) of patients underwent adrenalectomy without AVS guidance.

Then, a significant portion of procedures are non-diagnostic (see Table 4). In cases of unilateral sampling failure, useful information may still be gleaned. Pasternak et al. (Pasternak et al. 2016) showed 100% specificity and 50% sensitivity to be able to lateralize patients with unilateral data using the formula [(unilateral adrenal vein aldosterone÷cortisol)/(IVC aldosterone÷cortisol)]. A value > 5.5 accurately predicted ipsilateral production while values < 0.5 predicted contralateral production. Values in between contained cases of both bilateral and unilateral secretion. Similar high specificity but low sensitivity results have been shown in other studies (Lin et al. 2019).

AVS may lead to complications. While older publications describe a high (10%) rate of complications, including venous rupture, adrenal infarction and hypertensive crisis, subsequent publications have shown complications to be rare (Daunt 2005). In the largest published multicenter observational studies to date, the complication rate was 0.61% (16/2604) (Rossi et al. 2012). This decreasing rate of complications is likely due to the growing understanding that venography should be gentle and low volume. Catheter induced adrenal vein thrombosis may also occur if an occlusive catheter left in situ for a prolonged period, therefore, some recommend administration of IV heparin at the start of the procedure (Kahn and Angle 2010).

While cortisol is used both as a marker of adrenal vein selection and as a way to normalize aldosterone levels by accounting for dilution, cortisol can be pathologically co-secreted in a small percentage of aldosterone adenomas. This co-secretion may lead to false bilateral results as the corrected aldosterone level (A/C ratio) on the side of the co-secreting APA will be low. In cases where cortisol co-secretion is suspected, one may use another adrenal marker, such as metanepherines (Goupil et al. 2015).

Finally, the marked variety of protocols and indices leads to variable subtype classification, treatments and outcomes. In a study by Klein et al. (Kline et al. 2008), only 17% (11/63) patients would be classified the same across the all the various protocols and indices. Lethielleux et al. (Lethielleux et al. 2015) repeated a similar analysis by retrospectively analyzing data from 537 non-stimulated simultaneous AVS procedures. They found that there is a 4.5x difference in sample inadequacy using the most lenient (≥1.1) versus most strict (≥3) SI (4% (19/537) vs 18% (99/537)). They also found that while 58.2% (313/537) of patients would meet criteria for unilateral lateralization (with LI ≥ 2), only 25.8% (139/537) would be classified as unilateral utilizing the combined modified lateralization index [(Dominant side Aldosterone÷Cortisol ratio)/ (Inferior Vena Cava Aldosterone÷Cortisol)] ≥2 with concomitant CSI < 1 suggested by the group from the University of Queensland (Stowasser et al. 2001). Further research is needed to determine what protocol and indices should be used.

## Conclusions

PA is a common disease whose ill effects are beyond elevation of blood pressure. AVS is done to determine if aldosterone production is unilateral or bilateral. Surgery is generally preferred in the former while medical treatment is utilized in the latter. Non-diagnostic adrenal vein sampling can be minimized with proper provider training, experience and knowledge as well as selective use of rotational CT and real time rapid cortisol assays. Understanding the advantages and disadvantages of the wide variety of different protocols is important in optimizing AVS performance.

## Data Availability

Systematic review of published literature. All articles used cited in bibliography.

## Abbreviations

PA: Primary aldosteronism
ARR: Aldosterone to renin ratio
APA: Aldosterone producing adenoma
MR: Mineralocorticoid receptor
AVS: Adrenal vein sampling
CT: Computed tomography
MRI: Magnetic resonance imaging
LI: Lateralization index
ACTH: Adrenocorticotropic hormone
SI: Selectivity index
CSI: Contralateral suppression index
A/C ratio: Aldosterone to cortisol ratio
